# Effects of larvicidal and larval nutritional stresses on *Anopheles gambiae* development, survival and competence for *Plasmodium falciparum*

**DOI:** 10.1186/s13071-016-1514-5

**Published:** 2016-04-23

**Authors:** Amélie Vantaux, Issiaka Ouattarra, Thierry Lefèvre, Kounbobr Roch Dabiré

**Affiliations:** MIVEGEC (Maladies Infectieuses et Vecteurs: Ecologie, Génétique, Evolution et Contrôle), UMR IRD 224-CNRS 5290-Université de Montpellier, Montpellier, France; Institut de Recherche en Sciences de la Santé (IRSS), Bobo Dioulasso, Burkina Faso; Centre Muraz, Bobo Dioulasso, Burkina Faso

**Keywords:** *Bti*, Larvicide, Larval diet, Stress, Mosquito competence, Malaria parasite, *Plasmodium falciparum*

## Abstract

**Background:**

Many studies have shown that the environment in which larvae develop can influence adult characteristics with consequences for the transmission of pathogens. We investigated how two environmental stresses (larviciding and nutritional stress) interact to affect *Anopheles gambiae* (previously *An. gambiae* S molecular form) life history traits and its susceptibility for field isolates of its natural malaria agent *Plasmodium falciparum*.

**Methods:**

Larvae were reared in the presence or not of a sub-lethal concentration of larvicide and under a high and low food regimen. Development time, individual size, adult survival and competence for *P. falciparum* were assessed.

**Results:**

Individuals under low food regimen took more time to develop, had a lower development success and were smaller while there was no main effect of larvicide exposure on these traits. However, larvicide exposure impacted individual size in interaction with nutritional stress. Female survival was affected by the interaction between gametocytemia, parasite exposure and larval diet, as well as the interaction between gametocytemia, parasite exposure and larvicidal stress, and the interaction between gametocytemia, larvicidal exposure and larval diet. Among the 951 females dissected 7 days post-infection, 559 (58.78 %) harboured parasites. Parasite prevalence was significantly affected by the interaction between larvicidal stress and larval diet. Indeed, females under low food regimen had a higher prevalence than females under high food regimen and this difference was greater under larvicidal stress. The two stresses did not impact parasite intensity.

**Conclusions:**

We found that larval nutritional and larvicidal stresses affect mosquito life history traits in complex ways, which could greatly affect *P. falciparum* transmission. Further studies combining field-based trials on larvicide use and mosquito experimental infections would give a more accurate understanding of the effects of this vector control tool on malaria transmission.

**Electronic supplementary material:**

The online version of this article (doi:10.1186/s13071-016-1514-5) contains supplementary material, which is available to authorized users.

## Background

Mosquitoes are vectors of several deadly human diseases such as malaria, yellow fever, dengue fever and filariasis, resulting in worldwide morbidity and mortality. Close to half of the world population is at risk of contracting malaria, mainly in populations of sub-Saharan Africa, where children under 5 years of age and pregnant women are the most severely affected [1]. Despite all control efforts, around 600,000 people still die of malaria each year [1]. While current anti-malarial strategies have been successful in numerous countries, continuous emergence of mosquito insecticide resistance and parasite drug resistance, combined with the lack of an effective malaria vaccine, impede malaria control in areas of high transmission.

Current malaria vector control mostly relies on two main components: long-lasting insecticide treated bed nets and indoor residual spraying. However, they target only the adult stage, and individuals exhibiting endophilous and endophagous behaviours. In addition, the emergence and spread of insecticide resistance [2–5], as well as behavioral changes [6–8] threaten current malaria vector control programs. Thus, larval source management has received a renewed interest: targeting the immature stages could help reducing overall adult mosquito density [9, 10]. Larviciding, the use of substances to kill or inhibit the development of mosquito larvae, is one such source of larval management method. *Bacillus thuringiensis israelensis* (*Bti*) is a naturally occurring bacterium producing crystal toxin toxic to mosquito larvae and has been commercialised under various formulations [11]. *Bti* has shown promising results against many mosquito vectors [12–18]. While this kind of bio-pesticide is unlikely to pose any hazard to humans, its efficacy depends on regular application due to a short residual activity. *Bti* efficacy may also be difficult to maintain because *Bti* is sensitive to environmental characteristics such as vegetation and sun exposure, and breeding sites where applications are desired but may be difficult to identify [10].

Nutritional organic matter is also highly variable in larval habitats and can influence adult mosquito life history traits [19]. Food availability is particularly relevant in host-pathogen interactions as it affects both partners. First, it is an important determinant of host fitness, affecting key parameters of pathogen transmission such as host density and longevity [20, 21]. Secondly, nutritional stress can affect pathogen development in two opposing ways. On the one hand, food deprivation can hinder pathogen development through limited resource availability [22, 23]. On the other hand, food deprivation can enhance pathogen infection through the limited resources available for host immune responses [24, 25].

In malaria vectors, *An. gambiae* larvae provided with a small quantity of food had a lower melanisation capacity at the adult stage [24], potentially increasing their competence for pathogens. In contrast, other studies have shown that females that developed from nutritionally stressed *An. gambiae* and *An. stephensi* larvae had a decreased competence for *Plasmodium yoelii nigeriensis* and *P. yoelii yoelii*, respectively [26, 27].

In mosquito-borne diseases, epidemiological models generally assume that individual mosquitoes are equally likely to get and transmit the pathogen. However, many studies have shown that the environment in which larvae develop strongly determines adult characteristics influencing both vectorial capacity (i.e. the potential intensity of transmission by mosquitoes) [28–30] and vector competence (i.e. their ability to develop and transmit pathogens) [31, 32]. Therefore, environmental factors, such as sub-lethal doses of larvicides which harm but do not kill individuals, could greatly influence adult vectorial capacity and competence [33, 34].

To our knowledge, no study has yet explored the effects of both larvicidal and larval nutritional stresses on epidemiologically-relevant traits. In this study, we investigate how these two environmental stressors interact to affect *An. gambiae* (previously *An. gambiae* S molecular form; see [35]) life history traits including development time, body size, survival and competence for field isolates of its natural malaria agent *P. falciparum*.

## Methods

### Ethical approval

Ethical approval was obtained from the Centre Muraz Institutional Ethics Committee under agreement no. A003-2012/CE-CM. The protocol conforms to the Helsinki Declaration on ethical principles for medical research involving human subjects (version 2002) and informed written consent was obtained from all volunteers.

### Mosquitoes

Three to 5-day-old laboratory-reared females of *An. gambiae* were obtained from an outbred colony established in 2008. This colony is repeatedly replenished with F1 from wild-caught female mosquitoes collected in Kou Valley (11°23'14"N, 4°24'42"W), 30 km from Bobo Dioulasso, south-western Burkina Faso (West Africa). Females are identified by species-diagnostic PCR [36]. Mosquitoes were maintained under standard insectary conditions (27 ± 2 °C, 70 ± 5 % relative humidity, 12:12 Light: Dark photoperiod).

### Larval nutritional stress

Two experimental groups were set up: larvae reared under plentiful food condition and larvae reared under scarce food conditions based on the amounts of Tetramin® baby fish food provided daily (Table 1). The scarce food condition was intended to induce nutritional stress, while the plentiful food condition was intended to provide sufficient resources for a good larval development [24, 26, 37, 38]. We placed 300 first instar larvae in plastic trays (30 × 20.5 × 6.5 cm) containing 1 l of spring water. Food was distributed daily at the same hour. The larvae were counted every 2 days to adjust food quantities. The water was replaced every 2 days until the third larval stage. Pupae were transferred to plastic cups for emergence in a 30 × 30 × 30 cm cage and the adults were provided with a 5 % glucose solution on cotton wool pads. A total of 131 trays (45 high food and 86 low food) were used over four replicates. The scarce food condition was applied to more trays than the plentiful food condition to compensate for the higher mortality expected in nutritionally stressed larvae and still obtain enough mosquitoes for measurements of adult life history traits.

**Table 1:** Larval food quantity (mg/larva/day)

Larval stage	High food	Low food
1	0.075	0.025
2	0.1	0.05
3	0.2	0.75
4	0.3	0.1

### Larvicidal stress

First, we assessed the 50 (LC_50_) and the 90 (LC_90_) percent lethal concentrations of the granular formulation of *Bti* (VectoBac®; WG 3 000 UIT/mg; Lot number 60215-29-01; ValentBioScience Corporation, Illinois, USA) on *An. gambiae* larvae under the standard insectary conditions indicated above for eight concentrations: 0.1, 0.2, 0.3, 0.4, 0.5, 0.6, 0.7 and 0.8 mg/l. For each concentration 25 third stage larvae were placed in 150 ml of water. Larvae were not fed during the experiments, and all tests were run at standard insectary conditions. Larval mortality was recorded in both treated and control (without Vectobac®) trays after 24 h. Four replicates of each concentration and for each food treatment were run along with the control using distilled water. Secondly, we intended to expose larvae to sub-optimal Vectobac® concentration. Based on our results from LC_50_ and LC_90_ tests (see above), we chose to use the 0.1 mg/l concentration for both food regimens. This treatment was applied to 26 of the trays receiving plentiful food and to 51 of the trays receiving scarce food quantities. Overall, four experimental groups were obtained: (i) larvae reared with plentiful food and exposed to 0.1 mg/l of Vectobac® at the third instar; (ii) larvae reared with plentiful food only; (iii) larvae reared with scarce food and exposed to 0.1 mg/l of Vectobac® at the third instar, and (iv) larvae reared with scarce food only.

### Mosquito infection

Experimental infections were carried out as described in [39–41]. We used Direct Membrane Feeding Assays (DMFA) whereby gametocyte-infected blood is drawn from naturally-infected patients and from which mosquitoes feed through a membrane [42]. Gametocyte carriers were selected by examining thick blood smears from school children aged between 5 and 11 years from two villages in southwestern Burkina Faso (Dande and Soumousso, located 60 km north and 40 km southeast of Bobo-Dioulasso, respectively). To ensure that they would feed, the mosquitoes were only provided water for 24 h prior to being given access to a blood meal. As a negative control (non-infected mosquitoes), females were fed on the same blood in which the gametocytes were heat-inactivated. This heat-inactivation inhibits infection and does not affect the nutritive quality of the blood [43]. Heat-inactivation was achieved by placing the blood in a thermo-mixer and heated at 43 °C for 15 min and 900 rpm while the remaining blood was maintained at 37 °C. Three hundred μl of blood were distributed in membrane feeders maintained at 37 °C by water jackets. Four to 6 day-old female mosquitoes were allowed to feed for up to 2 h through a Parafilm® membrane. Fed females were sorted out and placed in new cages (30 × 30 × 30 cm). They had constant access to cotton wool pads imbibed with a 5 % glucose solution except for the subset of females used for the survival assays which did not receive glucose solution. Upon experimental infection, eight groups of mosquitoes were obtained: (i) those exposed to larvicidal, nutritional and infection stress; (ii) those exposed to larvicidal and nutritional stress; (iii) those exposed to larvicidal and infection stress; (iv) those exposed to nutritional and infection stress; (v) those exposed to larvicidal stress only; (vi) those exposed to nutritional stress only; (vii) those exposed to infection stress only; and (viii) unexposed control mosquitoes. Four replicates were performed with a total of six distinct gametocyte carriers (Table 2).

**Table 2:** Infection rates and intensity in females

Replicate	Gametocyte carrier	Gametocyte density (μl)	*Bti*	Larval diet	Infection rate ± 95 % CI	Infection intensity mean ± se
1	A	168	no	H	0.87 ± 0.17	26.7 ± 6.3
				L	0.7 ± 0.2	12.9 ± 2.7
			yes	H	0.48 ± 0.2	17.7 ± 7.9
				L	0.6 ± 0.25	14.3 ± 3.6
2	B	96	no	H	0.61 ± 0.12	5.3 ± 0.9
				L	0.62 ± 0.17	5.2 ± 1.4
			yes	H	0.75 ± 0.11	5.9 ± 0.7
				L	0.56 ± 0.23	5.8 ± 1.5
3	C	952	no	H	0.49 ± 0.11	38.7 ± 7.2
				L	0.63 ± 0.17	19.3 ± 4
			yes	H	0.54 ± 0.15	60.3 ± 9.3
				L	0.71 ± 0.1	35.6 ± 4.9
4	D	240	no	H	0.78 ± 0.13	33.1 ± 6.7
				L	0.82 ± 0.1	43.7 ± 5
			yes	H	0.65 ± 0.15	36.1 ± 8.4
				L	0.95 ± 0.06	54.2 ± 6.9
	E	80	no	H	0.1 ± 0.09	1.25 ± 0.2
				L	0.2 ± 0.12	2.12 ± 1.6
			yes	H	0.22 ± 0.13	1.3 ± 0.2
				L	0.17 ± 0.12	1.3 ± 0.3
	F	168	no	H	0.62 ± 0.15	21.1 ± 3.8
				L	0.52 ± 0.15	7.6 ± 1.6
			yes	H	0.71 ± 0.14	10.8 ± 1.9
				L	0.8 ± 0.12	10 ± 1.2

*Abbreviations*: L, low food mosquitoes; H, high food mosquitoes; *Bti*, *B. thuringiensis* var. *israelensis* larvicide; CI, Confidence Interval; *se,* standard error

### Measurements of mosquito life history traits

*Development time* was calculated as the duration from egg to emergence and was measured for a total of 17,747 individuals reared on four different replicates. *Wing length* was used as a surrogate of body size and was measured from the alula to the wing tip, excluding the scales [44]. One wing per individual was dissected for females 7 days post-blood meal and for males 3 days post-emergence. The wings were photographed using a dissecting microscope and measured with ImageJ software (Wayne Rasband, rsb.info.nih.gov/ij/). Wing length was compared on a subset of 1,755 individuals distributed over four replicates. *Survival* was assessed for an average of 15 females per experimental condition placed in 20 × 20 × 20 cm cages after they were fed blood. No glucose was provided but they had access to water *ad libitum*. Twice daily, we recorded the number of dead individuals. *Survival* data were obtained from a total of 648 individuals fed on the blood of three different gametocyte carriers from two replicates. *Competence*. Parasite prevalence (i.e. proportion of infected females) and intensity (i.e. number of *P. falciparum* oocysts in the midgut of infected females) were determined after dissection 7 days post-blood meal. The midguts were dissected in a 1 % Mercurochrome® stain and the presence and number of oocysts were determined under a light microscope. Four replicates with six different gametocyte carriers were conducted in which 951 individuals were used to determine oocyst prevalence. The oocyst intensity analysis was carried out on the 559 infected individuals. During dissection we discovered that some of our mosquitoes were infected with microsporidian parasites. Because microsporidians can affect *Plasmodium* development within mosquitoes [45], we accounted for this factor for mosquito competence analysis. The presence of microsporidia was observed across all treatment groups.

### Statistical analyses

*Lethal concentrations*: LC_50_ and LC_90_ of Vectobac were estimated using logistic regression in “*investr*” package [46] in R software environment. LC_50_ represents the probability of 50 % of the larvae dying and LC_90_ the probability of 90 % of the larvae dying. LC_50_ and LC_90_ were compared using the LC ratio tests wherein no differences are detected if the ratio contains 1 [47]. Ratios were estimated with the “*Comped*” function in “*drc*” package” [48]. *Development time* was analysed using Cox’s proportional hazard mixed effect models with sex, larval diet, larvicidal treatment and their interactions coded as fixed factors, and replicate as a random factor. Cox proportional hazard models were carried out with the “*Coxme*” function in the “*Coxme*” package [49]. *Wing length* was compared after log-transformation using a Linear Mixed Model with a Gaussian distribution (after confirming data normality). Larval diet, larvicidal treatment, sex and their interactions coded as fixed factors and replicate as a random factor. *Survival*: data were analysed using Cox’s proportional hazard mixed effects models with parasite exposure, larval diet, larvicidal treatment, gametocytemia and their interactions coded as fixed factors. Gametocyte carrier was nested in replicate and they were coded as random factors. Cox proportional hazard mixed effect models were carried out with the “*coxme*” function in the “*coxme*” package [49]. *Competence*: parasite prevalence and intensity were analysed using GLMMs with a binomial and a negative binomial error structure, respectively. In these GLMMs, larval diet, larvicidal treatment, gametocytemia, wing size, the presence of microsporidia and their interactions, were coded as fixed factors. Gametocyte carrier was nested in replicate and they were coded as random factors. Generalized Linear Mixed Models (GLMMs) with a binomial error and a logit link function were carried out with the “*glmer*” and the GLMMs with a negative binomial error structure were carried out with the “*glmer.nb*” function in “*lme4*” package [50]. For model selection, we used the stepwise removal of terms, followed by likelihood ratio tests (LRT). Term removals that significantly reduced explanatory power (*P* < 0.05) were retained in the minimal adequate model [51]. All analyses were performed in R v. 3.1.3 [52]. Results are presented as mean ± standard error (se) and proportion ± confidence interval (CI).

## Results

### Lethal concentrations

The LC_50_ and LC_90_ were 0.23 mg/l (95 % confidence interval (CI) = 0.2–0.26) and 0.52 mg/l (95 % CI = 0.48–0.56), respectively, for larvae exposed to high food quantities (hereafter, simply ‘high food’). The LC_50_ and LC_90_ were 0.14 mg/l (95 % CI: 0.10–0.16) and 0.36 mg/l (95 % CI = 0.33–0.40), respectively, for exposed to low food quantities (hereafter, simply ‘low food’) and were significantly different from the values of the high food larvae (LC_50_ ratio = 0.59 and LC_90_ ratio = 0.7, respectively).

### Development success and time

Overall, 3,650 out of the 5,700 larvae (64.03 ± 0.1 %) reared with plentiful of food only survived to the adult stage and 4,931 out of the 7,800 larvae (63.2 ± 0.01 %) reared with 0.1 mg/l of Vectobac® and plentiful of food survived to the adult stage. However, 3,720 out of the 10,500 larvae (35.4 ± 0.009 %) reared with scarce food only survived to the adult stage and 5,446 out of the 15,300 larvae (35.6 ± 0.008 %) reared with 0.1 mg/l of Vectobac® and scarce food survived to the adult stage. The high food larvae developed significantly faster than did their low food counterparts (11.82 ± 0.02 *vs* 14.42 ± 0.03 days, respectively; *χ*^*2*^ = 6505, *df* = 1, *P* < 0.0001). There were no significant differences in development time between larvae exposed or not to larvicidal stress (13.1 ± 0.03 *vs* 13.2 ± 0.02 days, respectively; *χ*^*2*^ = 2.2, *df* = 1, *P* = 0.14). There was no significant interaction between larval diet and larvicidal stress (*χ*^*2*^ = 2.8, *df* = 1, *P* = 0.09). See Additional file 1 for sex-specific results. The three-way interaction did not significantly impact development time (*χ*^*2*^ = 0.14, *df* = 1, *P* = 0.7).

### Wing size

**Fig. 1:**
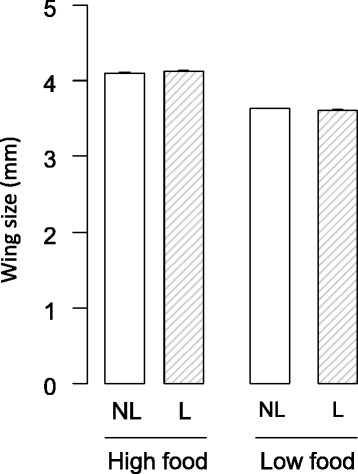
Effects of larvicidal and larval nutritional stresses on *Anopheles gambiae* wing sizes (mean ± standard error). *Abbreviations*: NL, no larvicidal stress; L, larvicidal stress

The mosquitoes exposed to the low food treatment as larvae were significantly smaller than were their high food counterparts (3.6 ± 0.007 *vs* 4.1 ± 0.008 mm; *χ*^*2*^ = 2286, *df* = 1, *P* < 0.0001). There was a significant interaction between larvicidal stress and nutritional stress (*χ*^*2*^ = 6.6, *df* = 1, *P* = 0.01): high food larvae exposed to larvicidal stress were bigger than the unexposed ones, while the reverse was true for low food larvae (Fig. 1). The larvicidal treatment did not significantly affect wing size (*χ*^*2*^ = 0.03, *df* = 1, *P* = 0.9). See Additional file 1 for sex-specific results. The three-way interaction did not significantly impact wing size (*χ*^*2*^ = 0.03, *df* = 1, *P* = 0.87).

### Survival

**Fig. 2:**
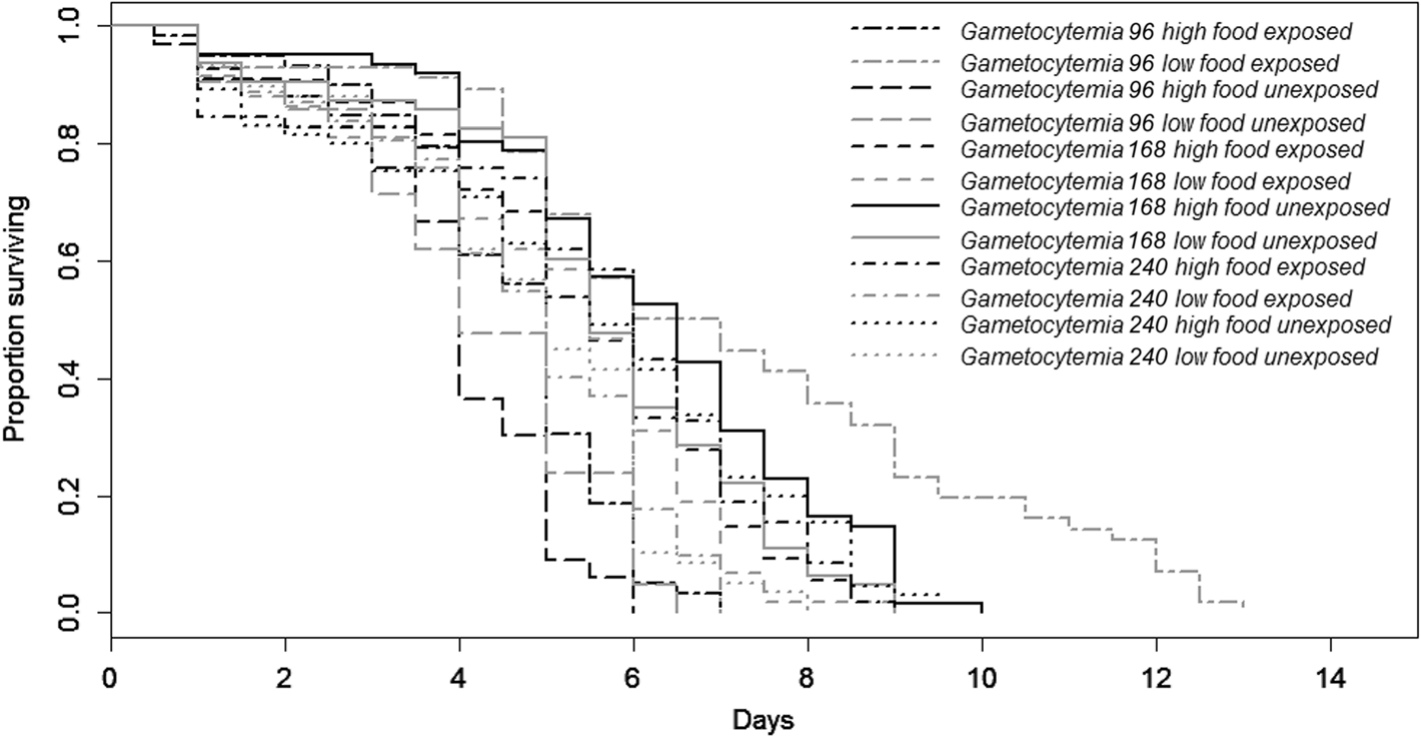
Effects of larval nutritional stress, parasite exposure and gametocytemia on survival rates

**Fig. 3:**
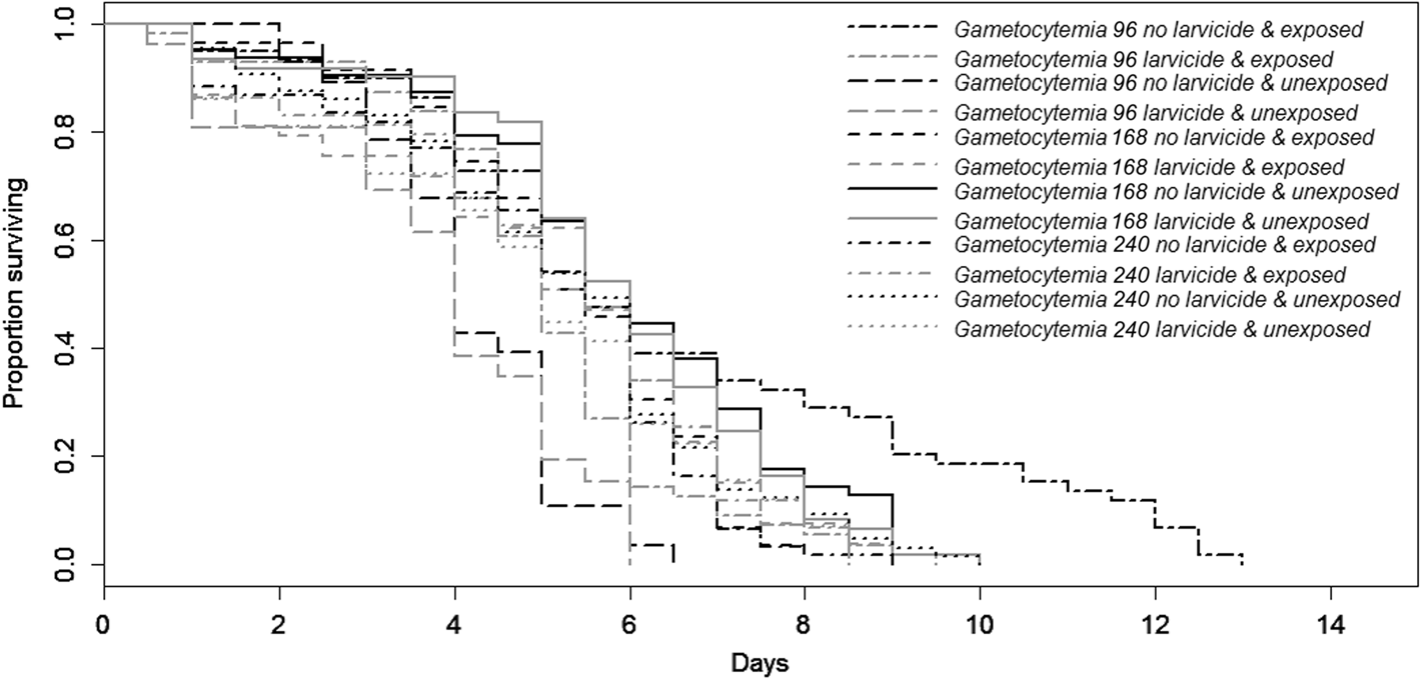
Effects of larvicidal stress, parasite exposure and gametocytemia on survival rates

**Fig. 4:**
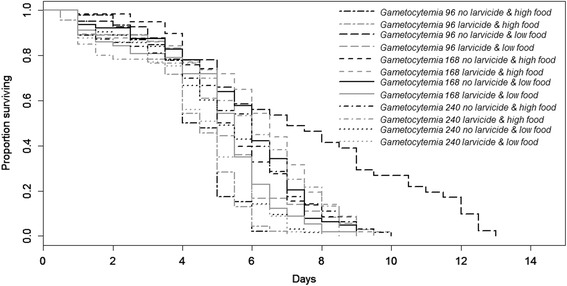
Effects of larval nutritional stress, larvicidal stress and gametocytemia on survival rates

Mosquito survival was significantly affected by larval diet (*Z* = 3.4, *P* = 0.0007, Fig. 2). In particular, low food females had a significantly better survival rate than high food ones (5.3 ± 0.1 *vs* 5.2 ± 0.1 days, see Additional file 2: Figure S3). Mosquito survival was significantly affected by gametocytemia (*Z* = 2.28, *P* = 0.02, Fig. 2). In particular, they had a better survival at low gametocytemias (gametocytemia 96: 5.21 ± 0.2 days; gametocytemia 168: 5.52 ± 0.1 days) than at high gametocytemia (gametocytemia 240: 5.04 ± 0.1 days). Larvicidal stress did not significantly affect mosquito survival (5.04 ± 0.1 *vs* 5.5 ± 0.1 days for larvicidal stressed and unstressed females, respectively; *Z* = 1.64, *P* = 0.1). Parasite exposure did not significantly affect mosquito survival (5.31 ± 0.1 *vs* 5.19 ± 0.1 days for exposed and unexposed females, respectively; *Z* = 1.66, *P* = 0.1). There was a significant interaction between larvicidal exposure and larval diet (*Z* = 3.42, *P* = 0.0006): when unexposed to larvicidal stress low food females had a higher survival rate than high food females (5.74 ± 0.2 *vs* 5.17 ± 0.15 days, respectively) whereas under larvicidal stress low food females had a lower survival rate than high food females (4.82 ± 0.2 *vs* 5.26 ± 0.2 days, respectively). There was a significant interaction between gametocytemia and larval diet (*Z* = 3.62, *P* = 0.0003, Fig. 2). In particular, for gametocytemia 96, survival rate was higher for low-food than for high food females (6.29 ± 0.4 *vs* 4.3 ± 0.1 days, respectively) whereas for gametocytemia 168 and 240, survival rates were higher for high food than for low food females (5.76 ± 0.2 *vs* 5.3 ± 0.2 days and 5.38 ± 0.2 *vs* 4.69 ± 0.2 days for gametocytemia 168 and 240, respectively). There was a significant interaction between larval diet and parasite exposure (*Z* = 3.31, *P* = 0.0009, Fig. 2). Indeed, high food females had a greater survival than low food females when unexposed to the parasite (5.35 ± 0.2 *vs* 5.02 ± 0.2 days for high and low food) whereas they had a lower survival when exposed to the parasite (5.09 ± 0.2 days *vs* 5.54 ± 0.2 days for high and low food, respectively). There was a significant interaction between gametocytemia and parasite exposure (*Z* = 2.1, *P* = 0.04): unexposed females had a lower survival rate at gametocytemia 96 than females exposed to the parasite (3.99 ± 0.2 days *vs* 5.78 ± 0.3 days, respectively), while they had a better survival at gametocytemia 168 (5.86 ± 0.2 *vs* 5.15 ± 0.2 days, respectively) or similar at gametocytemia 240 (5.05 ± 0.2 *vs* 5.03 ± 0.2 days, respectively). There was a significant three-way interaction between gametocytemia, parasite exposure and larval diet (*Z* = 2.66, *P* = 0.008, Fig. 2): low food females had a higher survival than high food females at gametocytemia 96 with a greater difference when exposed to the parasite, whereas high food females had a better survival rate than low food females at both gametocytemias 168 and 240 with a greater difference when exposed to the parasite for gametocytemia 240 only. There was a significant three-way interaction between gametocytemia, parasite exposure and larvicidal stress (*Z* = 2.14, *P* = 0.03, Fig. 3): females exposed to larvicidal stress had a lower survival than unstressed females both when exposed or not to the parasite at gametocytemia 96. The trend was similar although with a smaller difference at gametocytemia 168. However, females exposed to larvicidal stress had a lower survival than unstressed females when unexposed to the parasite, while females exposed to larvicidal stress had a greater survival than unstressed females when exposed to the parasite There was a significant three-way interaction between gametocytemia, larvicidal exposure and larval diet (*Z* = 2.36, *P* = 0.02, Fig. 4): survival was lower in larvicidal stressed females both in high food and low food females for the gametocytemia 96. For gametocytemias 168 and 240 survival was higher in females exposed to larvicidal stress compared to unstressed ones in high food females whereas the opposite was observed in low food females. The interaction between larvicidal exposure and gametocytemia (*Z* = 1.45, *P* = 0.15), the three-way interaction between larval diet, parasite exposure and larvicidal stress (*χ*^*2*^_1_ = 0.8, *df* = 1, *P* = 0.37), and the 4-way interaction (*χ*^*2*^ = 2.8, *df* = 1, *P* = 0.12) did not significantly impact mosquito survival rates.

### Competence

**Fig. 5:**
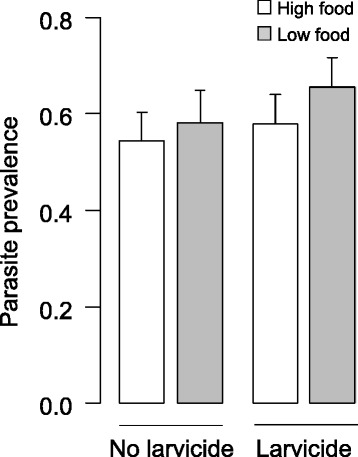
Effects of larval nutritional and larvicidal stresses on *P. falciparum* prevalence (± confidence interval)

Among the 951 females dissected 7 days post-infection, 559 (58.78 %) harboured parasites. The gametocyte densities in the blood samples, and the infection prevalence and intensities in the mosquitoes are provided in Table 2. Parasite prevalence was significantly affected by the presence of microsporidia (*χ*^*2*^ = 5.2, *df* = 1, *P* = 0.02) with females harbouring microsporidia having a significantly lower parasite prevalence than females with undetected microsporidia in their midgut (53.9 ± 0.04 *vs* 58.7 ± 0.04, respectively). Parasite prevalence was significantly affected by the interaction between larvicidal stress and larval diet (*χ*^*2*^ = 4.3, *df* = 1, *P* = 0.04; Fig. 5), with low food females having a higher prevalence than high food females and the difference was greater under larvicidal stress. No other significant effects were observed (see Additional file 1).

Infected low food mosquitoes did not harbour significantly more parasites than did infected high food mosquitoes (26.13 ± 1.94 *vs* 22.75 ± 1.92 oocysts; *χ*^*2*^ = 2.05, *df* = 1, *P* = 0.15). Likewise, the intensity of larvicidal-stressed mosquitoes was similar to that of unstressed mosquitoes (25.93 ± 2.03 *vs* 22.76 ± 1.8 oocysts; *χ*^*2*^ = 0.1, *df* = 1, *P* = 0.75). The presence of microsporidia negatively affected oocyst intensity (22.86 ± 2.22 in microsporidia-positive individuals *vs* 31.51 ± 2.07 oocysts in microsporidia-free individuals; *χ*^*2*^ = 631.2, *df* = 1, *P* < 0.0001). There was a negative relationship between individual size and parasite intensity (*χ*^*2*^ = 494.78, *df* = 1, *P* < 0.0001). No other significant effects were observed (see Additional file 1).

## Discussion

Environmental stressors are of great importance in vector-borne diseases as any alterations in adults traits could have crucial implications for transmission through changes in epidemiologically relevant traits such as longevity or vector competence [20, 21, 24, 26, 27, 34, 53–55]. In addition, multiple stressors can act synergistically, such as lower LC_50_ and LC_90_ in low food larvae compared to high food larvae suggesting that well-fed larvae were better able to cope with the larvicide.

Larval development was particularly affected by nutritional stress. Indeed, low food individuals took more time to develop, had a lower development success and were smaller. In addition, males took longer to develop than females but this difference was smaller in low food larvae compared to high food larvae. Interestingly, larvicidal stress decreased the difference in development time between males and females compared to unstressed larvae, suggesting that this stress tend to smooth out the development time differences between sexes. However, the larvicidal stress did not have as much impact as nutritional stress on larval development. From an ecological point of view, the increased developmental time in low food larvae may reduce the number of mosquito generations yearly and likely increase exposure to other stressors or risks such as competition, predation or drought which would also affect mosquito life history traits.

Overall, low food adults were smaller than high food adults. However, opposite effects were observed when these two groups were exposed to larvicidal stress. Indeed, in high food larvae, larvicidal stress increased adult size. On the other hand, in low food larvae, adult size was lower in larvae exposed to larvicide compared to unexposed individuals. One explanation could be that despite the fact that larvicidal stress did not affect development time, it selected individuals better able to cope with toxin damage induced by *Bti*. In high food larvae only bigger and likely more robust individuals survived, while in low food larvae, which were probably overall less fit, larvae feeding less and therefore less exposed to *Bti* toxins survived better, ending up with slightly smaller individuals overall. Small females often need two or three blood meals to be able to sustain the first gonotrophic cycle, as the first blood meal is used to replenish reserves [29, 56]. Therefore, females exposed to both stressors might be more inclined to have several blood meals thus increasing their chances of being infected and transmitting malaria [29, 56].

In mosquito-malaria parasite systems, host longevity is a particularly important epidemiological factor as it enters into estimates of vectorial capacity in a non-linear way. Indeed, *Plasmodium* parasites have a long development time in their vector before being transmissible [57], and only a few mosquitoes survive long enough to transmit them [58]. Consequently, any reduction in vector survival may impede transmission and conversely any increase in survival may considerably favour pathogen transmission [59]. Exposure to stresses was expected to reduce host survival. However, larvicidal stress had no effect on its own, and adult survival was higher in low food females compared to their high food counterparts. This unexpected result could be explained by an hormetic stress: exposure to mild stress induces protective mechanisms resulting in biologically beneficial effects [60]. However, more investigations are needed to explain this point.

Survival was affected by the interactions between nutritional and larvicidal stresses as well as parasite exposure and gametocytemia in various ways. Indeed, low food females had a higher survival than high food females at gametocytemia 96 with a greater difference when exposed to the parasite, whereas high food females had a better survival rate than low food females at both gametocytemias 168 and 240. Females exposed to larvicidal stress had a lower survival than unstressed females both when exposed or not to the parasite at gametocytemia 96. The trend was similar although with a smaller difference at gametocytemia 168. However, females exposed to larvicidal stress had a lower survival than unstressed females when unexposed to the parasite, while females exposed to larvicidal stress had a greater survival than unstressed females when exposed to the parasite. Survival was lower in larvicidal stressed females both in high food and low food females for the gametocytemia 96. For gametocytemia 168 and 240 survival was higher in females exposed to larvicidal stress compared to unstressed ones in high food females whereas the opposite was observed in low food females. These various differing trends and the strong variation in relation to gametocytemia might be due to the parasite’s genetic factors (e.g. infection intensity, multiplicity of infection) or to blood quality (e.g. composition, quantity). This study reinforces the point that evaluating survival rates is by no means simple and can depend on many factors.

Parasite prevalence was significantly affected by larval nutritional stress in interaction with larvicidal stress (Fig. 5). Indeed, low food females were significantly more infected than high food females, with even higher parasite prevalence and greater differences in larvicidal-stressed females compared to unstressed females. Thus females exposed to both larvicidal and nutritional stressors had the highest parasite prevalence which might be due to the host limited resources available for mounting an immune response [24, 25]. From a vector control perspective it implies that the application of sub-lethal dose of larvicide such as post-treatment or by difficulties to reach or evaluate fully larval breeding sites might result in a rebound in malaria transmission in low food settings. Indeed, the double stress would increase the number of infected females and the likelihood of malaria transmission.

Interestingly, parasite intensity was not impacted by larval nutritional and larvicidal stresses. Thus, these environmental stressors seem to impact host qualitative resistance, as measured by their ability to prevent infection, but not their quantitative resistance as measured by their ability to limit parasite development.

Parasite prevalence and intensity were decreased by the presence of microsporidia. Microsporidian parasites replicate throughout the mosquitoes life and negatively impact development, survival and fecundity as well as susceptibility to malaria parasites [61, 62]. This effect on malaria vector competence is likely due to priming of the mosquito’s immune system [61, 62].

We observed a negative relationship between parasite intensity and individual size which suggests that large females sustained a lower parasite load than small females. This is in contradiction to previous work [63]. One explanation could be that large females were better able to limit parasite development due to more energetic or immune resources available [24, 25].

## Conclusions

In conclusion, we found that larval nutritional and larvicidal stresses affect mosquito life history traits in complex ways, which could greatly affect malaria parasite transmission. Indeed, stresses negatively impacted development time or adult size or survival, while they increased mosquito competence for *Plasmodium falciparum*. Further studies combining field-based trials on larvicide use and mosquito experimental infections would give a more accurate understanding of the effects of this vector control tool on malaria transmission. Finally, differences in larval habitat quality could have crucial implications for the dynamics of malaria transmission. Only by considering the environmental conditions occurring in natural systems with the interplay of several stressors will we be able to fully comprehend natural mosquito-malaria parasite interactions and their impacts on malaria epidemiology.

## Additional files

Additional file 1: Supplementary results. (DOCX 13 kb) Additional file 2: Supplementary figures on development time (**Figure S1**), mosquito wing size (**Figure S2**) and mean survival time (**Figure S3**). (DOCX 271 kb)
