# Supplementary material for: Effects of larvicidal and larval nutritional stresses on Anopheles gambiae development, survival and competence for Plasmodium falciparum
Source: Parasit Vectors. 2016 Apr 23;9:226. doi: 10.1186/s13071-016-1514-5 (PMC4842262; doi:10.1186/s13071-016-1514-5)
Supplement: Additional file 1: — Supplementary results. (DOCX 13 kb) [file 13071_2016_1514_MOESM1_ESM.docx]

**Results**

*Development success and time* – The male to female sex ratio was 0.93 for the individuals reared with plentiful of food only, 0.98 for the individuals reared with Vectobac^®^ and plentiful of food, 0.85 for the individuals reared with scarce food only and 0.88 for the individuals reared with Vectobac^®^ and scarce food. The males developed significantly faster than did the females (12.99 ± 0.03 *vs.* 13.33 ± 0.03 days, respectively; X^2^_1_=86.5, P<0.0001). There was a significant interaction between larval diet and the sex of the mosquito (X^2^_1_=11.9, P=0.006): the difference in development time between males and females being higher under the high food treatment compared to the low food treatment (high food: 11.67 ± 0.02 *vs*. 11.96 ± 0.02 for males and females respectively; low food: 14.28 ± 0.04 *vs*. 14.54 ± 0.03 for males and females respectively, Fig. S1A). There was a significant interaction between larvicidal stress and the sex of the mosquito (X^2^_1_=6.5, P=0.01): the difference in development time between males and females being higher in individuals unexposed to larvicidal stress compared to exposed ones (no larvicidal stress: 12.9 ± 0.04 *vs*. 13.29 ± 0.04 for males and females respectively; larvicidal stress: 13.06 ± 0.03 *vs*. 13.35 ±0.03 for males and females respectively, Fig. S1B).

*Wing size* – The males were significantly smaller than were the females (3.76 ± 0.01 *vs.* 3.9 ± 0.01mm, respectively; X^2^_1_=310, P<0.0001). There was a significant interaction between larvicidal stress and the sex of the mosquito (X^2^_1_=7, P=0.008): the size difference between larvicidal stressed and unstressed individuals was bigger in males than in females (3.96 ± 0.1 and 3.94 ± 0.01 for larvicidal exposed and unexposed females respectively, and 3.77 ± 0.2 and 3.74 ± 0.2 for larvicidal exposed and unexposed males respectively, Fig. S2). There was no significant interaction between larval diet and the sex of the mosquito (X^2^_1_=0.007, P=0.9).

*Competence*

Parasite prevalence was not significantly affected by larval diet (*X*^2^_1_=2.4, P=0.12), wing size (*X*^2^_1_=0.08, P=0.77), larvicidal stress (*X*^2^_1_=0.7, P=0.41), gametocytemia (*X*^2^_1_=0.2, P=0.64), the interaction between larval diet and gametocytemia (*X*^2^_1_=2.1, P=0.15), between larvicidal stress and microsporidia presence (*X*^2^_1_=2.2, P=0.14), between gametocytemia and microsporidia presence (*X*^2^_1_=0.37, P=0.54), between gametocytemia and larvicidal stress (*X*^2^_1_=0.23, P=0.63), between microsporidia prevalence and larval diet (*X*^2^_1_=0.001, P=0.97), the three-way interaction between microsporidia prevalence, larvicidal stress and gametocytemia (*X*^2^_1_=0.95, P=0.33), between larval diet, larvicidal stress and gametocytemia (*X*^2^_1_=0.53, P=0.46), between larval diet, larvicidal stress and microsporidia prevalence (*X*^2^_1_=0.55, P=0.46), between larval diet, gametocytemia and microsporidia prevalence (*X*^2^_1_=2.87, P=0.09) nor the four-way interaction (*X*^2^_1_=0.86, P=0.35) significantly affected parasite prevalence.

Parasite intensity was not significantly affected by gametocytemia (*X*^2^_1_=1.7, P=0.19) or the interaction between larval diet and larvicidal stress (*X*^2^_1_=2.3, P=0.13) or between larvicidal stress and microsporidia presence (*X*^2^_1_=2.04, P=0.15), or between larval diet and gametocytemia (*X*^2^_1_=1.85, P=0.17), or between larval diet and microsporidia presence (*X*^2^_3_=0.4, P=0.52), or between microsporidia presence and gametocytemia (*X*^2^_1_=0.24, P=0.62), or between larvicidal stress and gametocytemia (*X*^2^_1_=0.05, P=0.82), or the three-way interaction between larval diet, larvicidal stress and gametocytemia (*X*^2^_1_=1.26, P=0.26), or between larvicidal stress, gametocytemia and microsporidia presence (*X*^2^_1_=0.2, P=0.65), or between larvicidal stress, larval diet and microsporidia presence (*X*^2^_1_=0.2, P=0.65), or between larval diet, gametocytemia and microsporidia presence (*X*^2^_1_=0.01, P=0.9), or by the four-way interaction (*X*^2^_1_=0.026, P=0.87).
