# Supplementary material for: Effects of larvicidal and larval nutritional stresses on Anopheles gambiae development, survival and competence for Plasmodium falciparum
Source: Parasit Vectors. 2016 Apr 23;9:226. doi: 10.1186/s13071-016-1514-5 (PMC4842262; doi:10.1186/s13071-016-1514-5)
Supplement: Additional file 2: — Supplementary figures on development time (Figure S1), mosquito wing size (Figure S2) and mean survival time (Figure S3). (DOCX 271 kb) [file 13071_2016_1514_MOESM2_ESM.docx]

**Additional file 2**

**Figure S1** Effects of (A) larval nutritional stress and (B) larvicidal stress on *Anopheles gambiae* males and females development time (mean ± se). F: females, M: males

**Figure S2** Effects of larvicidal stress and sex of the mosquitoes on *Anopheles gambiae* wing sizes (mean ± se). F: females, M: males

**Figure S3** Effects of (A) larval nutritional stress, parasite exposure and gametocytemia, (B) larvicidal stress, parasite exposure and gametocytemia, (C) larval nutritional stress, larvicidal stress and gametocytemia on mean survival time (±se). Numbers indicate gametocytemia values.
